# Social Isolation as a Mechanism Linking Sensory Impairment With Cognitive Functioning

**DOI:** 10.1093/geroni/igaa057.2095

**Published:** 2020-12-16

**Authors:** Jeremy Yorgason, Corinna Trujillo Tanner, Stephanie Richardson, Allison Burch, Brian Stagg, Melanie Hill

**Affiliations:** 1 Brigham Young University, provo, Utah, United States; 2 Brigham Young University, Provo, Utah, United States; 3 University of Utah, Salt Lake City, Utah, United States; 4 Brigham Young University, Orem, Utah, United States

## Abstract

Hearing and vision loss have been linked with cognitive decline in older adults. There may be various pathways through which sensory impairments impact cognitive functioning. Sensory impairments may lead individuals to be less socially connected, which may impact cognitive functioning due to less cognitive stimulation. As such, sensory impairments and social isolation may cascade to negatively impact cognitive functioning. Using data from 8,334 individuals aged 65-90+ in waves 6, 7, and 8 of the NHATS study, we estimated a longitudinal mediation structural equation model. Findings indicate that both self-reported vision and hearing impairment in wave 6 of NHATS were linked to concurrent cognitive functioning through social isolation. Only hearing impairment demonstrated longitudinal impact through social isolation across 2 and 3 waves. Findings suggest that medical professionals working with older adults with vision or hearing impairment should assess social isolation, as a point of intervention to maintain cognitive function.

